# Acute Truncal Ataxia After a Minor Head Trauma Revealing a Pediatric Cerebellar Pilocytic Astrocytoma

**DOI:** 10.7759/cureus.112091

**Published:** 2026-07-05

**Authors:** Connor Peeples, Chaitali Parikh, Melody L Milliron

**Affiliations:** 1 Emergency Medicine, Lake Erie College of Osteopathic Medicine, Erie, USA; 2 Emergency Medicine, Allegheny Health Network Saint Vincent Hospital, Erie, USA

**Keywords:** cerebellar pilocytic astrocytoma, minor head trauma, pediatric brain tumor, posterior fossa tumor, truncal ataxia

## Abstract

Pilocytic astrocytomas are low‑grade benign pediatric brain tumors that most commonly arise in the cerebellum and typically present with symptoms related to impaired coordination or increased intracranial pressure. We report the case of a three‑year‑old girl with no prior neurologic history who presented to the emergency department (ED) after a fall from a trampoline. Neurologic examination was notable for right‑sided truncal ataxia without additional focal weakness or sensory deficits. Non‑contrast computed tomography (CT) of the head demonstrated a hypodense lesion in the left cerebellum with trace hyperdensity. Magnetic resonance imaging (MRI) revealed a 3.5‑cm left cerebellar mass consistent with a pilocytic astrocytoma. The patient underwent surgical resection with no residual tumor on postoperative imaging and complete resolution of truncal ataxia symptoms on follow‑up.

## Introduction

Cerebellar pilocytic astrocytomas are low-grade benign central nervous system (CNS) tumors that comprise 10% of all intracranial pediatric neoplasms and 30% of pediatric posterior fossa tumors [1]. Although pilocytic astrocytomas can be found throughout the CNS, they commonly arise in the cerebellum [2]. Patients are aged 6-8 on average at diagnosis [3]. Pilocytic astrocytomas have a favorable prognosis with a 25-year survival rate of 90% [4]. Pilocytic astrocytomas respond well to surgery, particularly if the tumor is fully resected [5].

The cerebellum is responsible for balance and coordination; therefore, the signs and symptoms of cerebellar tumors arise from disruptions to those functions. The three most common symptoms of pilocytic astrocytomas are headache, vomiting, and gait disturbance, and the three most common signs are papilledema, ataxia, and nystagmus [1]. Symptoms of headache and vomiting may occur as well due to increased intracranial pressure.

Overall, it is rare for pediatric brain tumors such as pilocytic astrocytomas to present without symptoms and be found incidentally due to imaging for another reason. About 0.04-5.7% of pediatric patients undergoing imaging will be diagnosed with an incidental brain tumor [5]. Furthermore, it is even more rare for traumatic events to precipitate symptoms of cerebellar tumors that were previously asymptomatic [5].

Cerebellar (posterior fossa) tumors can produce significant peritumoral edema or hemorrhage. Due to the confined anatomy of the posterior fossa, even focal lesions may cause brainstem compression, hydrocephalus, and bilateral neurological deficits. Edema and injury to cerebellar pathways may result in bilateral symptoms such as ataxia, cranial nerve dysfunction, and altered level of consciousness [6]. Although cerebellar tumors typically present with symptoms ipsilateral to the lesion due to cerebellar pathway anatomy, clinical laterality is not always predictable. This is especially true when edema or hemorrhage creates a mass effect within the restricted space of the posterior fossa. These changes may occur as part of the tumor's natural course or after trauma to the region involved [6]. Brain tumors may undergo intratumoral hemorrhage either spontaneously or after trauma, leading to rapid expansion, increased mass effect, and acute clinical deterioration. Case reports suggest that even minor trauma can trigger tumor rupture or bleeding with subsequent worsening of neurologic or systemic symptoms [7,8].

Therefore, we report the case of a three-year-old girl who was previously asymptomatic and presented to the emergency department (ED) with acute right-sided truncal ataxia after a fall from a trampoline. She was later diagnosed with a left cerebellar pilocytic astrocytoma based on imaging and surgical pathology findings.

## Case presentation

A three-year-old girl with an unremarkable past medical or surgical history presented to the ED following a fall from a trampoline. She previously had two visits to the ED and subsequent outpatient pediatric follow-up appointments prior to the ED visit. Her most recent primary care visit was approximately one week before her fall, and there were no neurologic signs or symptoms on evaluation. Her family stated she and her sibling were playing on a trampoline when she struck her head against the netting. She had no loss of consciousness or cephalohematoma reported. She presented to the ED with head and neck pain. The family noted the patient was leaning towards the right side when walking.

Upon arrival at the ED, neurologic exam findings included an alert and oriented patient with intact cranial nerve testing and no focal deficits present. Pupils were round and reactive to light. Coordination testing (appendicular) showed that finger-to-nose and heel-to-shin testing were intact bilaterally without dysmetria. When instructing the patient to sit upright in the C-collar, the patient immediately leaned to the right side and was unable to sit up straight on examination.

Her gait assessment showed subtle rightward postural deviation. Otherwise, gait was normal. Her reflexes were noted as 2/4 triceps and biceps bilaterally and 2/4 patellar reflexes bilaterally. Babinski was not pathologically present. Her motor strength was 5/5 in the upper and lower extremities. Her sensory exam showed fine touch and pinprick intact throughout. There was also mild cervical spine tenderness upon examination; therefore, a cervical collar was placed. Her truncal ataxia became more apparent after the placement of the cervical collar. There was no weakness or numbness present.

**Figure 1:**
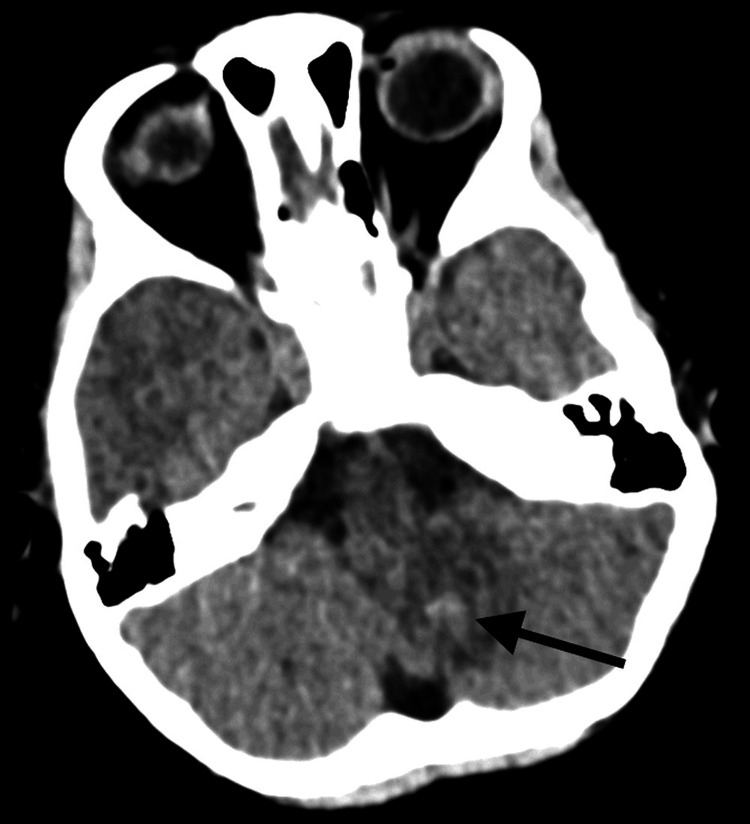
Axial slice of the computed tomography of the head without contrast The arrow notes a hypodensity in the left cerebellum with trace hyperdensity along the inferior aspect raising suspicion for a cerebellar mass with possible hemorrhage.

**Figure 2:**
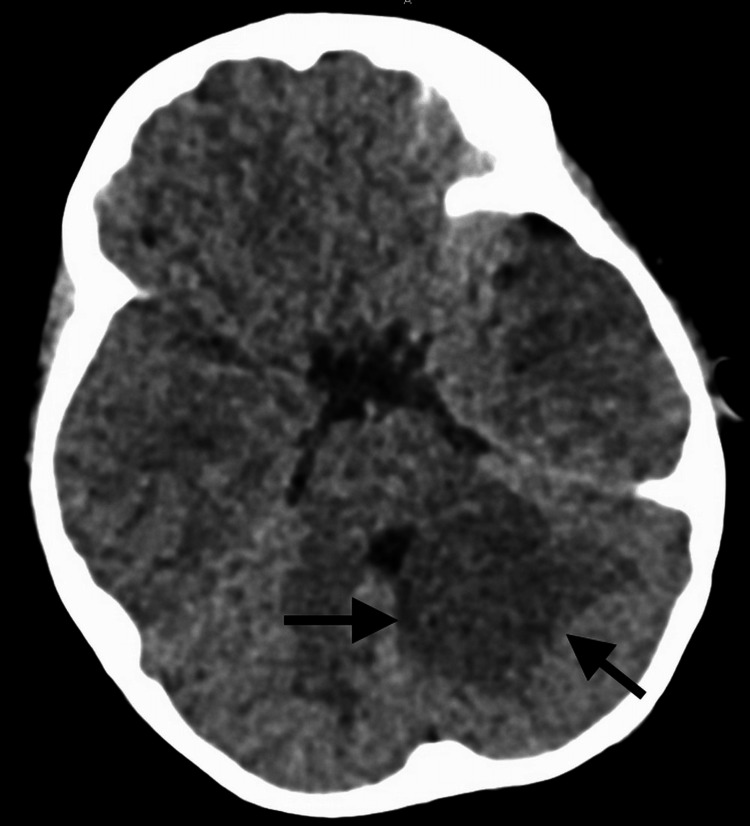
Axial slice of the computed tomography of the head without contrast The arrows note a suspected cerebellar mass.

Computed tomography (CT) of the head without contrast (Figure 1) showed a hypodensity in the left cerebellum with trace hyperdensity along its inferior aspect that raised suspicion for a cerebellar mass. The hyperdensity that was observed alongside the hypodensity was thought to possibly represent a hemorrhage secondary to the trauma. Partial effacement of the fourth ventricle without evidence for supratentorial hydrocephalus was also noted on the CT (Figure 2). The patient was transferred to a pediatric tertiary care facility for definitive care and management.

Unfortunately, the authors do not have access to the magnetic resonance imaging (MRI) or the full operative report from the outside facility. Therefore, the authors are able to report only from chart review on follow-up evaluation with the patient's local pediatrician. MRI with and without contrast at that facility showed a left cerebellar mass measuring 3.5 cm with imaging features that were most consistent with either a pilocytic or a pilomyxoid astrocytoma. Local mass effect with partial effacement of the fourth ventricle was also observed. Operative intervention was performed, and postoperative imaging showed stable and healed postoperative changes from the resection of her tumor. Minimal postoperative gliosis was observed in the left inferior cerebellum. There was no evidence of residual or recurrent tumor. The subsequent pathology reports from the tertiary care facility identified the lesion as a pilocytic astrocytoma. There were also no neurologic findings at follow-up, and her ataxia had resolved. She remained asymptomatic during an unrelated ED visit two years later.

## Discussion

Pilocytic astrocytomas are common cerebellar glial tumors in children and adolescents. Approximately 15.6% of all brain tumors and 5.4% of all gliomas are pilocytic astrocytomas [9]. Patients present with symptoms such as dysmetria, truncal ataxia, gait instability, dizziness, and weakness, as the cerebellum plays a major role in coordination and balance [1]. Patients may also present with headaches and vomiting due to increased intracranial pressure. Most patients have a good prognosis after resection [10,11].

Cerebellar symptoms are typically ipsilateral due to the cerebellar pathway organization. However, in this case, the patient had right-sided cerebellar findings despite a left-sided cerebellar lesion. One possible hypothesis regards the swelling and suspected hemorrhage surrounding the tumor on imaging. Within the confined space of the posterior fossa, associated mass effect can alter the clinical presentation and sometimes produce symptoms on either side [6]. Another speculated mechanism relates to the tumor's proximity to the midline. Given its location being near the cerebellar vermis, it is possible that the left-sided swelling observed on CT produced sufficient mass effect to displace adjacent structures within the right cerebellar hemisphere, resulting in the disruption of pathways involved in truncal coordination. [12]. An alternative hypothetical explanation is that the tumor was not responsible for the patient's symptoms at all. As the patient sustained impact to the right posterior aspect of the skull, it is possible that a traumatic right cerebellar contusion or injury of another structure, such as the vestibular cochlear complex, produced the observed right-sided imbalance symptoms.

The mechanism underlying the emergence of tumor-related symptoms following trauma is also a unique and noteworthy aspect of this case that warrants further exploration. This patient was asymptomatic until she fell from the trampoline and had an acute onset of cerebellar symptoms, particularly ataxia. Although all brain tumors are asymptomatic until presentation, it is exceedingly rare for symptoms to be provoked in the setting of minor trauma. One prior case report of a seven-year-old boy diagnosed with a cerebellar tumor mentioned that he presented with dizziness after falling in gym class [13]. However, the patient's family noted in hindsight that he had experienced headaches and dizziness for about four months prior to his fall. Therefore, cerebellar and mass effect symptoms were present before his traumatic event, in contrast to our patient. However, it can be difficult to determine prior history in a three-year-old, as they may have difficulty conveying symptoms like headaches and dizziness. For this reason, she could have had symptoms before the incident and been unable to voice them.

Trauma is known to precipitate hemorrhage and edema surrounding brain tumors as these lesions often contain malformed, fragile vasculature [6,7]. In this case with our patient, one speculative mechanism is that a relatively minor impact triggered a disproportionate hemorrhagic or edematous response, amplified by the delicate and disorganized structure of the neoplasm [6,7]. A similar finding occurred in an additional case where a cerebellar dermoid cyst had hemorrhaged and was found incidentally after imaging following head trauma [14]. Another hypothesis is that the tumor did not contribute to the cerebellar symptoms at all, as trauma may also produce cerebellar symptoms independent of a cerebellar tumor. In this case, imaging confirmed the presence of a cerebellar mass, leading us to speculate that the tumor was the most likely contributor to the patient's presentation. Although trauma-induced hemorrhage or edema within the tumor represents a plausible explanation for our patient's presentation, the absence of complete imaging correlation prevents definitive determination of causality and makes these explanations a hypothesis.

A limitation of this case is that the authors did not have access to the original MRI studies or the complete operative and pathology reports from the tertiary pediatric care center to which the patient was transferred. As a result, radiologic and operative findings were obtained from follow-up documentation of the reports rather than direct review of the source materials. The absence of direct imaging review and complete operative documentation limits the strength of the radiologic-pathologic conclusions that can be drawn from this case.

Another limitation of this case is the communication challenges inherent to evaluating toddlers. Determining what symptoms a young child is experiencing, as well as the precise onset of those symptoms, can be difficult. Toddlers may be unable to recognize or adequately describe subtle neurologic abnormalities such as mild truncal ataxia, dizziness, or headaches. These communication limitations can also reduce the accuracy of the patient's history by making it more difficult to ascertain the child's current symptoms and their severity. As a result, it is possible that mild symptoms were present before the inciting event but went unrecognized by the child or were not reported to caregivers. This limitation may make it difficult to establish the exact sequence and timing of symptom development and, consequently, to determine the relationship between the traumatic event and the patient's clinical presentation.

Overall, the asymptomatic nature of this patient's cerebellar mass prior to her fall, followed by the onset of cerebellar symptoms after a traumatic event, makes this a reportable case and provides an important educational opportunity regarding the potential for neurologic symptom emergence or clinical deterioration of underlying cranial tumor pathology following trauma. This case also highlights that it may be difficult to get a reliable preinjury history in younger patients, as there may have been subtle unreported symptoms prior to her fall. However, the absence of parents noting a gait or ambulation difficulty prior to her fall supports our hypothesis of an acute change after her seemingly minor fall.

## Conclusions

This case highlights an uncommon presentation of a cerebellar pilocytic astrocytoma identified after a seemingly minor head trauma. Cerebellar tumors typically present with progressive neurologic symptoms such as ataxia. However, an acute onset of truncal ataxia after trauma should prompt evaluation for an underlying structural lesion even when the mechanism appears to be low risk.

It also adds to the literature suggesting that tumors may be prone to hemorrhage, edema, or further injury after a seemingly minor trauma, leading to mass effect in the region of the skull in which they are located. This represents a plausible mechanism for what may have occurred in this patient. This case report underscores the importance of a broad differential diagnosis and obtaining appropriate imaging in pediatric patients with acute cerebellar findings. Early recognition and surgical intervention can result in excellent neurologic outcomes, as demonstrated in this patient.
